# Supplementary material for: Lipid Headgroup Charge and Acyl Chain Composition Modulate Closure of Bacterial β-Barrel Channels
Source: Int J Mol Sci. 2019 Feb 5;20(3):674. doi: 10.3390/ijms20030674 (PMC6386941; doi:10.3390/ijms20030674)
Supplement: Supplementary file 1 [file ijms-20-00674-s001.pdf]

Lipid Headgroup Charge and Acyl Chain Composition Modulate Closure of Bacterial β-Barrel Channels

Supplementary materials:

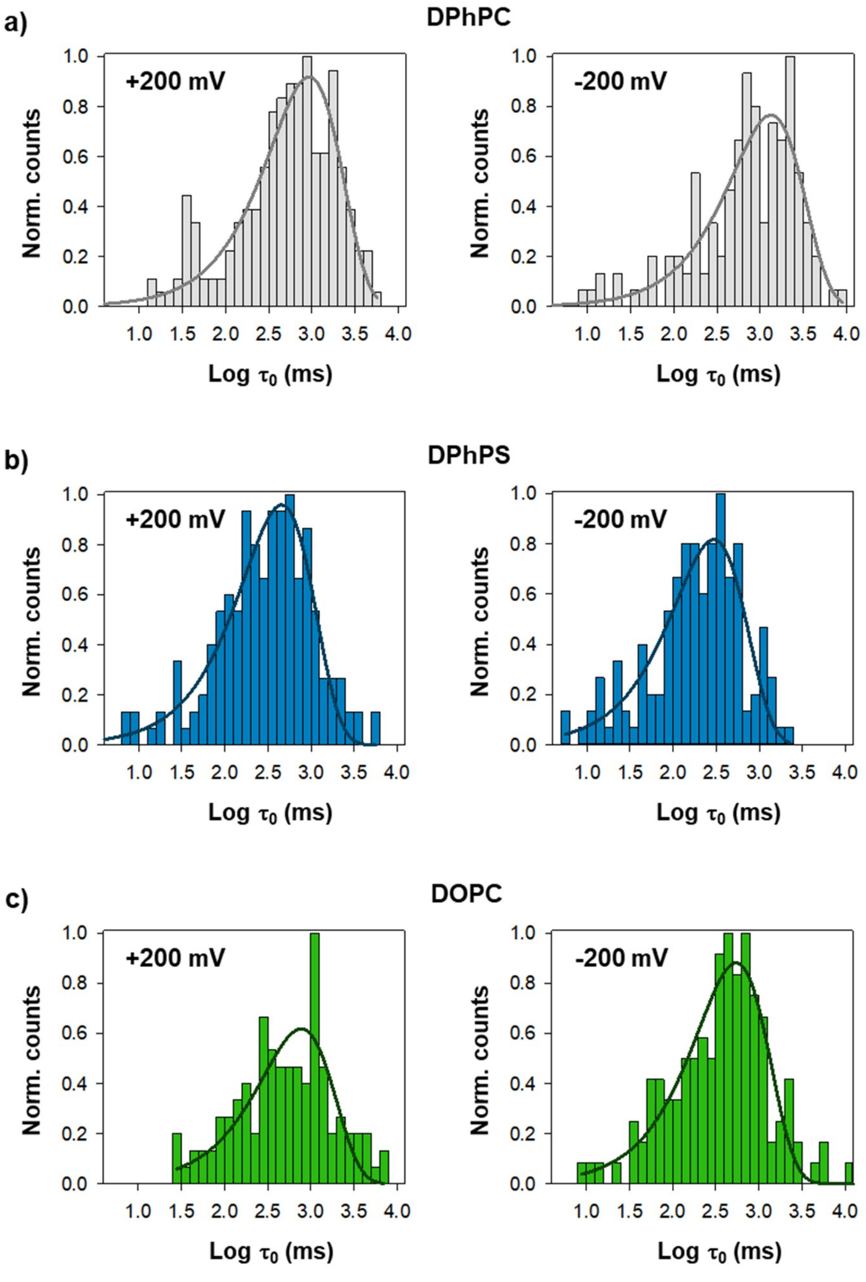

Figure S1. Individual view of the histograms shown in Figures 3 and 4. Logarithmically binned histograms of the time the channel spends in the open conformation when inserted in membranes composed of DPhPC **(a)**, DPhPS **(b)** or DPhPC/DOPC (1/1, labelled as DOPC) **(c)**, under a positive (*left panels*) or a negative (*right panels*) applied voltage. Solid lines are exponential fittings.

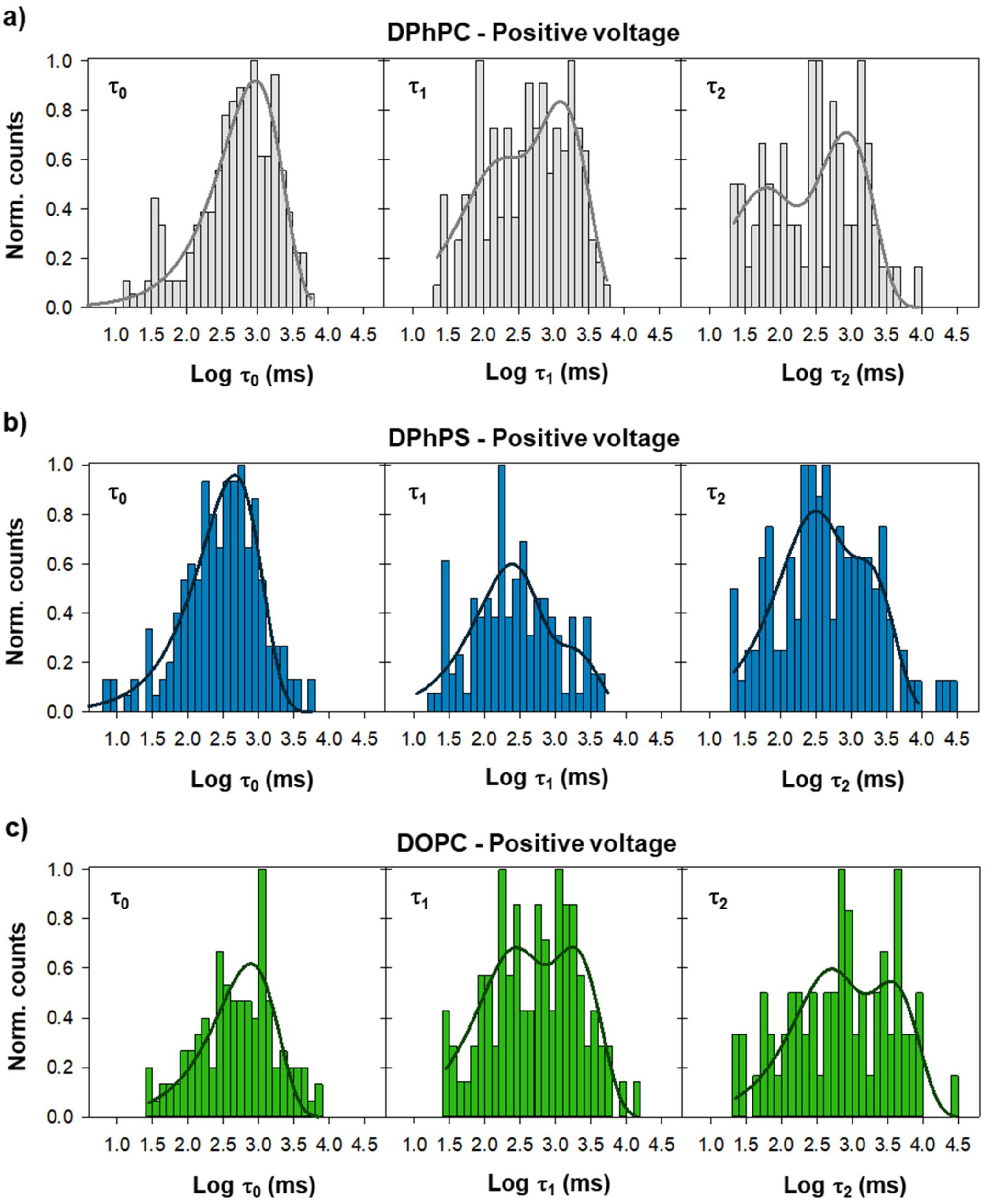

Figure S2. Individual view of the histograms shown in Figure5 for positive voltages. Logarithmically binned histograms of the time the channel spends before closing the first (τ_0), second (τ_1), or third (τ_2) monomers under a positive applied voltage when inserted membranes composed of DPhPC **(a)**, DPhPS **(b)**, or DPhPC/DOPC (1/1) **(c)**. Solid lines are exponential fittings with one (τ_0) or two (τ_1, τ_2) terms.

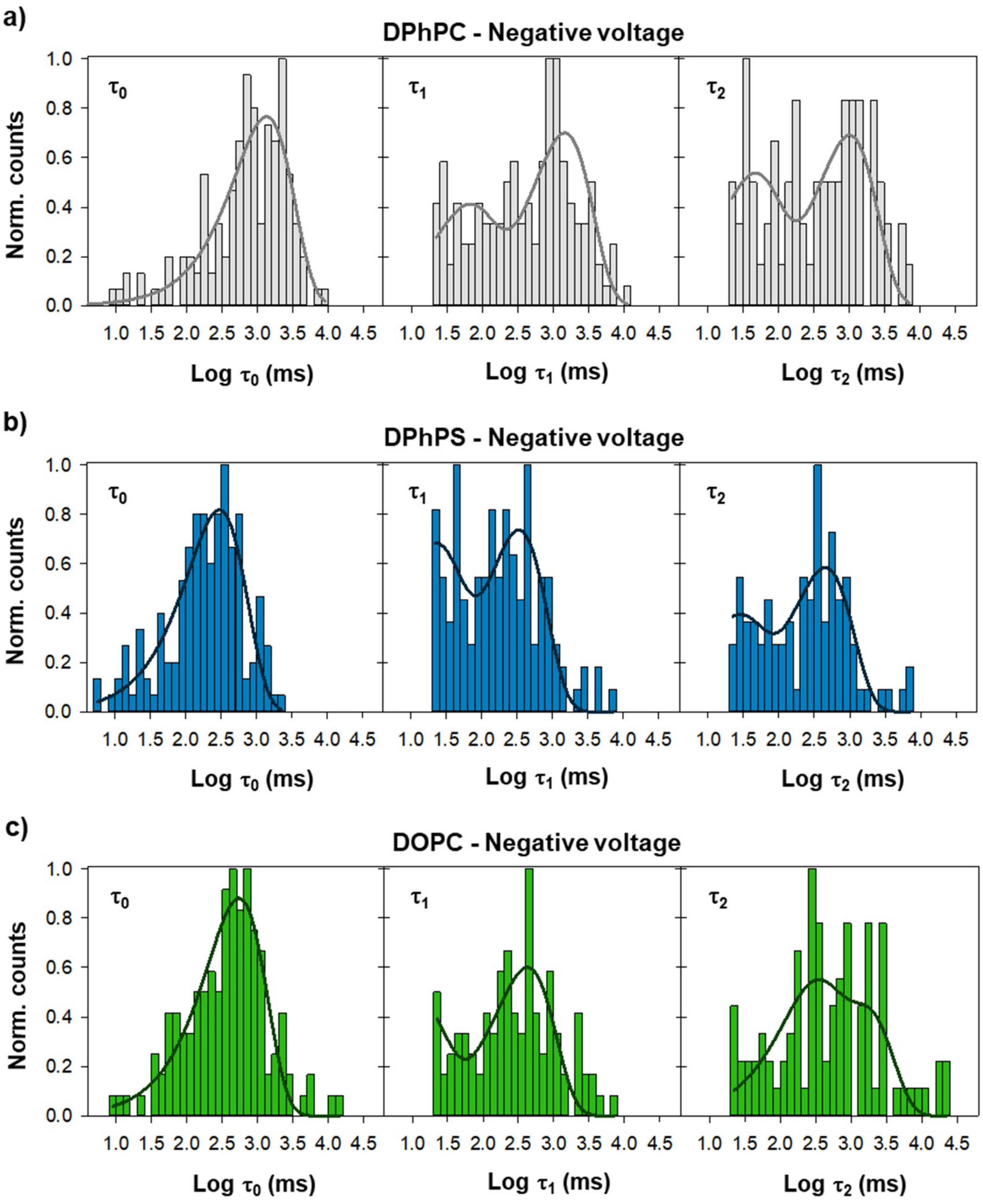

Figure S3. Individual view of the histograms shown in Figure5 for negative voltages. Logarithmically binned histograms of the time the channel spends before closing the first (τ_0), second (τ_1), or third (τ_2) monomers under a negative applied voltage when inserted membranes composed of DPhPC **(a)**, DPhPS **(b)**, or DPhPC/DOPC (1/1) **(c)**. Solid lines are exponential fittings with one (τ_0) or two (τ_1, τ_2) terms.

Statistical tests for significance in Figures 3c and 4c

All 6 experimental conditions (DPhPC, DPhPS, DPhPC/DOPC lipids with ±200 mV applied voltage) were checked together for significance with a one-way ANOVA. After the test stated that there is a statistically significant difference ($p < 0.001$) in the mean values among the treatment groups, a Holm-Sidak *post hoc* test was used for pair-wise comparison. Data were checked for normality and homoscedasticity with Shapiro-Wilk and Levene tests, respectively.

These are the results obtained:

ONE-WAY ANOVA

**Group Name**	**N**	**Missing**	**Mean**	**Std. Dev.**	**SEM**
DPhPC +200mV	3	0	941.967	10.894	6.289
DPhPC -200mV	4	0	1329.814	100.162	50.081
DPhPS +200mV	4	0	476.277	73.482	36.741
DPhPS -200mV	4	0	296.858	31.006	15.503
DOPC +200mV	4	0	856.023	97.995	48.997
DOPC -200mV	4	0	554.001	15.223	7.611

**Source of Variation**	**DF**	**SS**	**MS**	**F**	**P**
Between Groups	5	2768940.107	553788.021	119.287	<0.001
Residual	17	78921.980	4642.469		
Total	22	2847862.087			

DF = Degrees of freedom; SS = Sum of squares; MS = Mean square.

The differences in the mean values among the treatment groups are greater than would be expected by chance; there is a statistically significant difference (P = <0.001).

Power of performed test with alpha = 0.050: 1.000

All Pairwise Multiple Comparison Procedures (Holm-Sidak method):
Overall significance level = 0.05

Comparisons for factor:

**Comparison**	**Diff of Means**	**t**	**P**	**P<0.050**
DPhPC -200mV vs. DPhPS -200mV	1032.956	21.440	<0.001	Yes
DPhPC -200mV vs. DPhPS +200mV	853.537	17.716	<0.001	Yes
DPhPC -200mV vs. DOPC -200mV	775.813	16.103	<0.001	Yes

DPhPC +200mV vs. DPhPS -200mV	645.109	12.397	<0.001	Yes
DOPC +200mV vs. DPhPS -200mV	559.165	11.606	<0.001	Yes
DPhPC -200mV vs. DOPC +200mV	473.790	9.834	<0.001	Yes
DPhPC +200mV vs. DPhPS +200mV	465.690	8.949	<0.001	Yes
DOPC +200mV vs. DPhPS +200mV	379.746	7.882	<0.001	Yes
DPhPC +200mV vs. DOPC -200mV	387.966	7.455	<0.001	Yes
DPhPC -200mV vs. DPhPC +200mV	387.847	7.453	<0.001	Yes
DOPC +200mV vs. DOPC -200mV	302.022	6.269	<0.001	Yes
DOPC -200mV vs. DPhPS -200mV	257.143	5.337	<0.001	Yes
DPhPS +200mV vs. DPhPS -200mV	179.419	3.724	0.005	Yes
DPhPC +200mV vs. DOPC +200mV	85.944	1.652	0.220	No
DOPC -200mV vs. DPhPS +200mV	77.724	1.613	0.125	No

SHAPIRO-WILK TEST FOR NORMALITY:

DPhPC +200mV:	W-Statistic = 0.840	P = 0.214	Passed
DPhPC -200mV:	W-Statistic = 0.881	P = 0.341	Passed
DPhPS +200mV:	W-Statistic = 0.824	P = 0.152	Passed
DPhPS -200mV:	W-Statistic = 0.956	P = 0.755	Passed
DOPC +200mV:	W-Statistic = 0.901	P = 0.438	Passed
DOPC -200mV:	W-Statistic = 0.805	P = 0.112	Passed

A test that fails indicates that the data varies significantly from the pattern expected if the data was drawn from a population with a normal distribution.
A test that passes indicates that the data matches the pattern expected if the data was drawn from a population with a normal distribution.

LEVENE TEST FOR HOMOSCEDASTICITY

Equal Variance Test: Passed (P = 0.284)

A two-way ANOVA would have also been appropriate, in this case assuming that we have two separate factors affecting our experimental data: lipid and voltage polarity. The results in terms of significance between pairs are the same for the two designs.

TWO-WAY ANOVA

**Source of Variation**	**DF**	**SS**	**MS**	**F**	**P**
Voltage	1	5532.559	5532.559	1.192	0.290
Lipid	2	2077077.150	1038538.575	223.704	<0.001
Voltage x Lipid	2	489147.939	244573.970	52.682	<0.001
Residual	17	78921.980	4642.469		
Total	22	2847862.087	129448.277		

The effect of different levels of Voltage depends on what level of Lipid is present. There is a statistically significant interaction between Voltage and Lipid. (P = <0.001)

Power of performed test with alpha = 0.0500: for Voltage : 0.0666
Power of performed test with alpha = 0.0500: for Lipid : 1.000
Power of performed test with alpha = 0.0500: for Voltage x Lipid : 1.000

Least square means for Voltage :

**Group**	**Mean**	**SEM**
Positive V	758.089	20.733
Negative V	726.891	19.669

Least square means for Lipid :

**Group**	**Mean**	**SEM**
DPhPC	1135.890	26.020
DPhPS	386.567	24.090
DOPC	705.012	24.090

Least square means for Voltage x Lipid :

**Group**	**Mean**	**SEM**
Positive V x DPhPC	941.967	39.338
Positive V x DPhPS	476.277	34.068
Positive V x DOPC	856.023	34.068
Negative V x DPhPC	1329.814	34.068
Negative V x DPhPS	296.858	34.068
Negative V x DOPC	554.001	34.068

All Pairwise Multiple Comparison Procedures (Holm-Sidak method):
Overall significance level = 0.05

Comparisons for factor: Voltage

Comparison	Diff of Means	t	P	P<0.050
Positive V vs. Negative V	31.198	1.092	0.290	No

Comparisons for factor: Lipid

Comparison	Diff of Means	t	P	P<0.050
DPhPC vs. DPhPS	749.323	21.132	<0.001	Yes
DPhPC vs. DOPC	430.878	12.151	<0.001	Yes
DOPC vs. DPhPS	318.445	9.347	<0.001	Yes

Comparisons for factor: Lipid within Positive V

Comparison	Diff of Means	t	P	P<0.05
DPhPC vs. DPhPS	465.690	8.949	<0.001	Yes
DOPC vs. DPhPS	379.746	7.882	<0.001	Yes
DPhPC vs. DOPC	85.944	1.652	0.117	No

Comparisons for factor: Lipid within Negative V

Comparison	Diff of Means	t	P	P<0.05
DPhPC vs. DPhPS	1032.956	21.440	<0.001	Yes
DPhPC vs. DOPC	775.813	16.103	<0.001	Yes
DOPC vs. DPhPS	257.143	5.337	<0.001	Yes

Comparisons for factor: Voltage within DPhPC

Comparison	Diff of Means	t	P	P<0.05
Negative V vs. Positive V	387.847	7.453	<0.001	Yes

Comparisons for factor: Voltage within DPhPS

Comparison	Diff of Means	t	P	P<0.05
Positive V vs. Negative V	179.419	3.724	0.002	Yes

Comparisons for factor: Voltage within DOPC

Comparison	Diff of Means	t	P	P<0.05
Positive V vs. Negative V	302.022	6.269	<0.001	Yes
